# Nurses’ Practice Environment and Their Job Satisfaction: A Study on Nurses Caring for Older Adults in Shanghai

**DOI:** 10.1371/journal.pone.0138035

**Published:** 2015-09-18

**Authors:** Ying Wang, Weizhen Dong, Kristen Mauk, Peiying Li, Jin Wan, Guang Yang, Lyuying Fang, Wan Huan, Chun Chen, Mo Hao

**Affiliations:** 1 Research Institute of Health Development Strategies, Fudan University, Shanghai, China; 2 Munk School of Global Affairs, University of Toronto, 315 Bloor Street West (at the Observatory) Toronto, Ontario, Canada M5S 0A7; 3 College of Nursing and Health Professions, Valparaiso University, Valparaiso, Indiana, United States of America; 4 Scholl of Nursing, Hangzhou Vocational and Technical College, Hangzhou, Zhejiang, China; 5 College of Humanities and Management, Wenzhou Medical College, Zhejiang, China; Federal University of Rio de Janeiro, BRAZIL

## Abstract

**Aim:**

To examine the job satisfaction of nurses who are caring for older adults in healthcare settings in Shanghai, and to explore the underlying factors in order to explain and predict nurses’ job satisfaction.

**Background:**

China has the largest elderly population in the world, and its population is aging rapidly. Studies on job satisfaction of nurses providing care for the elderly in China can help to identify problem areas and develop strategies for the improvement of nurses’ working conditions. However, to date, this subject matter has not been thoroughly studied in the Chinese context. Previous studies in other countries show that many factors impact nurses’ job satisfaction, with the practice environment being a critical factor. There is a serious nursing shortage in China, especially in the big cities such as Shanghai. Given the increasing care demand of the aging population, learning about the job satisfaction level among nurses who are caring for older adults can provide essential information to help attract and retain nurses in this specialty area.

**Methods:**

A cross-sectional survey was conducted among 444 nurses in 22 elderly care institutions in Shanghai. The Chinese version of the Index of Work Satisfaction (IWS) and the Nursing Practice Environment Scale were instruments used. Inferential statistical tests used to analyze the data included Spearman correlation analysis, one-way analysis of variance, and hierarchical regression tests.

**Results:**

The average overall IWS (part B) score was 135.21 ± 19.34. Personality, job and organizational characteristics were found to be the most influential factors, and the practice environment was identified as having the strongest impact on job satisfaction (Beta = 0.494).

**Conclusion:**

Job satisfaction level among nurses who are caring for older adults in Shanghai is moderate, but the data suggest that this could be greatly increased if the nursing practice environment was improved.

## Introduction

Job satisfaction is defined as all the feelings an individual has about his/her job [1]. Many studies indicate that nurses’ job satisfaction is a key contributor to nursing absenteeism, burnout, turnover, and intention to quit [2, 3]; and that low job satisfaction among nurses is one of the most important reasons for the current shortage of nurses worldwide [4]. According to China's sixth national population census data bulletin, the Chinese population had reached 1.34 billion by 2010, with 8.87% of them aged 65 and over [5]. Shanghai is one of China’s most rapidly aging areas, with a consistently higher aging rate than the national average [6]. By the end of 2010, there were 233.0 (10.12%) thousand people aged 65 year or older in Shanghai [5].

In addition to comprising a large percentage of the population, older adults are far more vulnerable to chronic illness and require more health care services than other populations. In 2011, the number of partially or totally disabled older individuals in China was 33 million, and is expected to reach 40 million by 2015 [7]. Moreover, due to the One Child Policy, China tends to have smaller families with fewer children. Therefore, the availability of family members to provide care and support their older parents will likely decrease. Institutional and community care is expected to become an important option.

Shanghai, like the rest of China, is currently facing severe challenges within its eldercare nursing system. Since 1999, the Shanghai government has increased the construction of elderly care institutions, including increasing the number of elderly care facilities and strengthening the nursing staff’s training. However, according to Yang (2011), there is a significant shortage of 19,594 nurses among Shanghai’s eldercare nursing staff. This eldercare nursing staff shortage is a concern that is urgently in need of a solution by the Shanghai government.

Several studies [8, 9], mainly conducted outside China, suggest that improving nurses’ job satisfaction might be a solution to the nursing shortage problem. Therefore, the purpose of this study was to examine the job satisfaction of nurses who are caring for older adults in Shanghai, and explore the main factors that contribute to their job satisfaction, in order to develop strategies for maintaining a stable eldercare nursing professionals and meet the ever-increasing eldercare demand in China.

A considerable number of studies have shown that a variety of factors affect nurses’ job satisfaction, such as personality, organizational, and other job factors. Specifically, job satisfaction is influenced by personality factors including age [10], gender [4], marital status [11], education level [12], and working experience and tenure in nursing [13]; and organizational factors including size [14], geographical location [15], type of institution [16], and the nursing practice environment [10]; and job factors including workload [17] and stress [18]. Among all factors, practice environment has been identified as a key organizational factor [19], which has a stronger relationship with job satisfaction than any other organizational or personal factor [20].

Most of the studies in this area have largely focused on the job satisfaction issue among general nurses, although some have focused on particular nursing groups, such as community nurses [21], new graduate nurses [22], emergency nurses [23], psychiatric nurses [24], and ICU nurses [25]. Moreover, nurses' job satisfaction research has been ongoing for over 70 years [10, 15], but there is little in-depth research in this area in China, and especially a lack of research among nurses in elderly care institutions. Thus, this study is both timely as well as long overdue.

## Materials and Methods

### Sample

The Chinese eldercare system currently consists of two parts: home care and institutional care. There are two systems (the social welfare system and the health care system), that provide institutional care for elders [26]. Specifically, the social welfare system mainly offers custodial care, whereas intermediate care includes government and private-sector run senior housing and homes for the aged [27]; the health care system mostly provides professional care and transitional (sub-acute) care include nursing homes, community healthcare service centers (CHSCs), rehabilitation hospitals, geriatric hospitals, and emergency, geriatric, and rehabilitation departments in general hospitals. Since nurses, especially registered nurses (RNs) and licensed practical nurses (LPNs), are mainly concentrated within the health care system, this study focuses on job satisfaction among nurses in elderly care institutions.

Additionally, as a recent study shows, in Shanghai, 78.5% of old people select nursing homes and CHSCs for inpatient care, instead of hospitals [28]. Up to 95.9% of hospitalized patients in nursing homes and CHSCs are over 60 years old [29]. Therefore this study focuses on the nurses among nursing homes and CHSCs who are providing the majority of professional nursing services for older adults in Shanghai, China. According to the Shanghai Health Information Center and Shanghai Health Bureau, there were 210 CHSCs and 61 nursing homes–including 17 primary nursing homes (PNH), and 54 medical-institution-affiliated nursing homes (MIANH)- by the end of 2010 (Yang 2011).

This study employed a cross-sectional design to investigate nurses’ job satisfaction among Shanghai’s sample of nursing homes and CHSCs in August, 2011. First, 18 districts of Shanghai were divided into three levels based on the three socioeconomic levels: high, medium and low; random sampling was then conducted for two districts from each level (specifically, high level districts: Pudong and Changning district; medium level districts: Hongkou and Putuo; low level districts: Jinshan and Chongming). The number of nursing homes differs across districts according to the CHSCs setting regulation [30], issued by China’s ministry of health, but the number of CHSCs are relatively stable in China. Two CHSCs were randomly selected from each district; then, two nursing homes were randomly selected by alphabetical order of institutional name, including PNH and MIANH. The comparability of these institutions was ensured because the selected nursing homes and corresponding CHSCs have the same level of care and are located within three blocks of one another. Overall, 22 institutions participated in this study. Only one nursing home was qualified in the districts of Chongming and Hongkou, which are at medium and low socioeconomic level respectively.

### Participants

All RNs and LPNs who provide eldercare services in the sample-institutions voluntarily participated in the study. The participants’ inclusion criteria were based on the National Database of Nursing Quality Indicators (NDNQI 2003). RNs and LPNs who worked in nursing homes or the CHSCs and who spent at least 50 percent of their working hours on eldercare, and those who had been working there for at least 3 months were eligible to participate. In total, 444 nurses from the 22 eldercare institutions participated in the study.

### Data collection and instruments

The organizational characteristics data (type, size, and geographical location) were collected from the Shanghai Health Bureau. An anonymous survey with questionnaire was conducted to obtain information relating to the nurses’ demographic characteristics, such as age, gender, marital status, education level, professional status, and duration of nursing experience. The survey adopted several validated questionnaires from previous research.

#### Index of Work Satisfaction (IWS)

The IWS is a two-part (A & B) multidimensional instrument. In order to simplify the process, only Part B was employed in the study, since it is a commonly accepted tool in assessing job satisfaction among nurses [31]. Part B of the IWS is comprised of 44 items, each with a 7-point Likert-type scale:1 = strongly disagree; 7 = strongly agree). The scale was designed to measure six components of job satisfaction: pay, autonomy, task requirements, organizational policies, interaction and professional status [32].

Data from Part B of the IWS was analyzed using the procedures stipulated in the IWS scoring manual, with higher scores indicating greater job satisfaction. Mean scores of between 2.5 and 5.5 are believed to indicate moderate job satisfaction [32]. Part B of the IWS is reliable and valid with Cronbach’s alpha coefficients ranging from 0.35 to 0.90, and a total scale reliability of 0.82 to 0.90 [10].

#### Practice Environment Scale of the Nursing Work Index (PES-NWI)

This tool is considered a valid measure of the nursing practice environment and consists of a 4-point Likert-type 31-item scale, which is composed of five subscales that measure: (1) nurses’ participation in hospital affairs, (2) nursing foundations for quality care, (3) nursing managers’ ability; leadership and support of nurses, (4) staffing and resource adequacy, and (5) collegial nurse-physician relations. Each subscale is indicative of fundamental domain with respect to supporting professional nursing practice [19]. The total PES-NWI score is calculated as the mean of the five subscales scores. A mean subscale score of less than 2.5 represents a disagreement with the subscale measure in the current job situation whereas a mean subscale score above 2.5 represents agreement [33]. Coefficients for subscale internal consistency range from 0.71 to 0.84, with a reported Cronbach’s alpha of 0.82 [19].

#### Instrument translation

This study adopted the Chinese version of the IWS. It was translated and modified according to the Chinese context. The notable difference between the two versions is that the Chinese version employs a 5-point Likert-type items rather than the 7-point one. The reliability and validity scores of the Chinese version are reported as 0.835 and 0.918 respectively [34]. This study has also adopted the Chinese version of the PES-NWI [35], and reported reliability and validity scores of this version as 0.835 and 0.918, respectively.

### Ethical considerations

Ethics approval for the project was received from the Research Ethics Committee of Fudan University. The participants were assured that participation in the study was voluntary, with the return of the completed questionnaire as an act of consent to participate by the individual respondent. Respondents were assured of anonymity.

### Data analysis

The topic of this study is the job satisfaction of nurses who caring for older adults, which touches upon issues such as stress, workload, and the type of nursing employment unit, nurses’ personal characteristics and institutions’ organizational characteristics (including the nursing practice environment).

Data were analyzed using the Statistical Package for the Social Sciences (SPSS®), English version 17.0. The cut-off for significance was defined as a two-tailed *p*-value of <0.05. Data were summarized using descriptive statistics (mean ± SD, frequencies). The job satisfaction variables were classified using one-way analysis of variance (ANOVA). The associations between job satisfaction and practice environment were examined using Spearman’s correlation. Hierarchical regression analysis was performed to determine the relative and overall contribution of independent variables (personal characteristics, organizational characteristics, and practice environment) on job satisfaction. In the analysis, we used a forward stepwise method, comprising 3 steps. At each step, if results were significant we reported the following descriptors and statistics: B, Beta, P, R^2^, R^2^-changes, F, and significance value.

## Results

### Personal Characteristics

The personal characteristics of the respondents are shown in Table 1. All of the respondents were women (n = 444) and their average age was 31.27 ± 8.73, with more than a half of the respondents between the ages of 20 and 30 (n = 234, 52.70%). With regard to education, 22.30% had a Master’s or higher degree, 20.05% had a Bachelor’s degree, 38.51% had a college diploma, and 19.14% had a technical nursing school or lower level of education. Most of them were nurse and senior nurses (n = 339, 76.35%), while less than 5% held a chief nurse title (the highest level of nurses in China). The mean duration of their experience in nursing was 9.99 ± 8.50 years, and 56.31% of the respondents had worked in elderly care facilities for fewer than 9 years (n = 250).

**Table 1:** Descriptive characteristics and job satisfaction of the nursing samples and ANOVA analysis results.

Variables	Number	Percent (%)	IWS mean score(Part B)		ANOVAP
			*Mean*	*SD*	value
**Gender**					
Female	444	100.00	3.07	0.44	
**Age(years)**					0.0342*
< = 20	17	3.83	3.34	0.49	
20~30	234	52.70	3.09	0.45	
30~40	114	25.68	3.00	0.39	
40~50	62	13.96	3.06	0.46	
>50	17	3.83	3.10	0.43	
**Marital status**					0.0258*
Single/Widow/divorced	164	36.94	3.13	0.44	
Married	280	63.06	3.04	0.44	
**Education level**					<0.0001**
Technical nursing school diploma or lower	85	19.14	3.01	0.39	
College degree	171	38.51	2.98	0.40	
Bachelor degree	89	20.05	3.12	0.50	
Master degree or above	99	22.30	3.26	0.44	
**Professional status**					<0.0001**
Nurse and Senior Nurse	339	76.35	3.13	0.42	
Nurse-in-Charge	54	12.16	2.90	0.42	
Assistant Chief Senior Nurse	35	7.88	2.82	0.48	
Chief Senior Nurse	16	3.60	3.02	0.53	
**Duration of experience in nursing (years)**					0.0658
0~9	250	56.31	3.12	0.46	
9~19	118	26.58	3.03	0.38	
19~29	54	12.16	2.96	0.45	
>30	22	4.95	3.09	0.39	
**Type of institution**				-0.2301	<0.0001**
PNH	120	27.03	3.33	0.44	
MIANHs	123	27.70	2.96	0.43	
Community health service center	201	45.27	2.99	0.38	
**Geographic location**					<0.0001**
City center	210	47.30	2.98	0.43	
Suburb	137	30.86	3.27	0.45	
Exurb	97	21.85	3.01	0.36	
**Size** (Bed number)					<0.0001**
50-	67	15.09	3.01	0.42	
50–100	82	18.47	2.94	0.38	
100–500	175	39.41	2.98	0.40	
500+	120	27.03	3.33	0.44	
**Practice environment level**					<0.0001**
Unfavorable	31	6.98	2.55	0.51	
Mixed	90	20.27	2.75	0.29	
Favorable	323	72.75	3.21	0.38	

**P<0.01

*P<0.05

### Organizational Characteristics

The organizational characteristics of the study are shown in Table 1. The respondents were from three types of institutions: PNH, n = 120 (27.03%); MIANH, n = 123 (27.70%); and CHSCs, n = 201 (45.27%). Nearly half of them were working in the city center (n = 210, 47.30%) at the time of the study, and 66.44% of the respondents worked in large scale eldercare institutions with over 100 beds (n = 295).

### Practice Environment

Table 2 shows that the mean scores on the PES-NWI were 2.97, which indicated the practice environment was considered favorable overall. The most favorable aspect of the practice environment was the collegial nature of the nurse-physician relationship (subscale score of 3.27 ± 0.51), whereas the least favorable aspect was the adequacy of staffing and resources (subscale score of 2.64 ± 0.76).

**Table 2:** Descriptive statistics of nurses' responses to IWS and PES-NWI instrument.

	*Job satisfaction mean score(Part B)*							*Practice Environment score*					
	Interaction	Status	Autonomy	Organization-al policies	Task requirements	Pay	Total	Nurse participation in hospital affairs	Nursing foundations for quality care	Nursing manager ability	Staffing and resource adequacy	Collegial nurse physician relation	PES-NWIscore
**Mean**	3.78	3.23	3.20	3.04	2.81	1.85	3.07	2.69	3.19	3.07	2.64	3.27	2.97
**Std. Dev**.	0.54	0.62	0.61	0.71	0.52	0.78	0.44	0.58	0.50	0.59	0.76	0.61	0.51
**Max**	4.90	5.00	4.88	4.71	4.17	4.33	4.27	4.00	4.00	4.00	4.00	4.00	4.00
**Min**	1.40	1.29	1.25	1.00	1.00	1.00	1.59	0.98	0.80	0.96	0.80	0.80	1.02

Based on the favorability categories [36], the respondents’ scores indicated that there were more favorable than unfavorable ratings overall: (1) “unfavorable” (if scores were above 2.5 on zero or one subscale) n = 31, 6.98%, “mixed” (if scores were above 2.5 on 2 or 3 subscales) n = 90, 20.27%, and “favorable” (if scores were above 2.5 on 4 or 5 subscales) n = 323, 72.75%.

### Job satisfaction

#### Level of satisfaction

Results for level of job satisfaction are shown in Table 2. The average overall mean IWS score (part B) was135.21 ± 19.34. The mean subscale score (based on the Likert-type responses) was 3.07 ± 0.44, with subscale scores ranging from 1.85 to 3.78. Because the Chinese version of IWS uses 5-point and not 7-point items, the mean score was adjusted accordingly. The resulting value of 4.30 suggests moderate levels of job satisfaction. The highest level of satisfaction was observed on the interaction subscale (3.78 ± 0.54), whereas the lowest was observed for the pay subscale (1.85 ± 0.78).

#### Analyzing nurses’ job satisfaction according to personal and organizational characteristics

One-way ANOVAs were conducted to analyze nurses’ job satisfaction, and the results are given in Table 1. Age (P = 0.0342), marital status (P = 0.0258), education level (P<0.0001), professional status (P<0.0001), type of institution (P<0.0001), geographic location (P<0.0001), size (P<0.0001), and the practice environment (P<0.0001) were significantly related to nurses’ job satisfaction, whereas the duration of experience in nursing was not.

The study found that unmarried nurses under the age of 20 tended to have greater job satisfaction than those who are older and married, and the nurses with the highest levels of education (Master’s degree or above) showed greater job satisfaction than those with lower levels of education. Further, senior nurses and nurses were more likely to be satisfied with their jobs than those associate chief nurses or chief nurses. Institutionally, nurses who worked in the PNH were more satisfied than those who worked in MIANH or in CHSCs. Geographically, nurses who worked in the institutions that were located in ex-urban and suburban were more satisfied than those who were working in the city center. The nurses’ job satisfaction level was higher in larger scale eldercare institutions (>500 beds) than in smaller institutions (<500 beds). Nurses who worked in more favorable nursing practice environments tended to be more satisfied with their jobs than those who were working in unfavorable or mixed environments.

### Correlation between job satisfaction and practice environment

Prior to inferential tests, histograms and scatter plots were generated to examine if the data were abnormally distributed. Spearman’s r was computed as a screening tool to test for correlation between job satisfaction and the practice environment.

There was a significant strong positive correlation between job satisfaction and the nursing practice environment (r_s_ = 0.6601, P < 0.001). Except for the pay subscale, the majority of job satisfaction subscales were significantly correlated with the nursing practice environment subscale, with correlation coefficients ranging from 0.1259 (for pay correlated with staffing and resource adequacy) to 0.6917 (for organizational policies correlated with nurse participation in hospital affairs) (Table 3).

**Table 3:** Correlation analysis for job satisfaction and practice environment.

Variables	*Interaction*	*Professional status*	*Autonomy*	*Organizational policies*	*Task requirements*	*Pay*	*IWS part B mean score*
**Nurse participation in hospital affairs**	0.4503**	0.3333**	0.5638**	0.6917**	0.2757**	0.2383**	0.6224**
**Nursing foundations for quality care**	0.4799**	0.3660**	0.4552**	0.5106**	0.2517**	0.0753	0.5211**
**Nursing manager ability**	0.5174**	0.3409**	0.5007**	0.5410**	0.2413**	0.0871	0.5466**
**Staffing and resource adequacy**	0.3686**	0.3751**	0.4683**	0.5177**	0.3956**	0.1259**	0.5218**
**Collegial nurse physician relation**	0.6671**	0.3611**	0.4083**	0.3944**	0.2610**	0.1100*	0.5400**
**PES-NWI score**	0.5909**	0.4124**	0.5764**	0.6362**	0.3513**	0.1524**	0.6601**

**P<0.01

*P<0.05

### Predictors of job satisfaction

In order to determine the relationship and overall contribution of the variables to job satisfaction, a stepwise hierarchical regression analysis was performed (Table 4). Nurses’ job satisfaction was entered as the dependent variable in the regression equation. In step 1, the nurses’ demographic factors accounted for a small amount of the variance (R-squared = 0.080). In step 2, the institutional factors explained an additional 4.1% of the variance. In the final step, the nursing practice environment (PES-NWI score) was accounted for a further 22.0% of the variance in job satisfaction. After the three steps, more than a third of the variance in job satisfaction could be explained by the nursing practice environment (R^2^ = 0.341, Beta = 0.494, P < 0.001).

**Table 4:** Results of Hierarchical Regression Analysis.

*Step*	*Variable entered*	*B*	*Beta*	*P*	*R Square*	*R Square Change*	*F*	*Sig*
Step 1					0.080	0.080	7.568	<0.001
	**Personal Characteristics**							
	Education level	-0.079	-0.187	0.001				
	Professional status	0.087	0.156	0.001				
Step 2					0.121	0.041	7.496	<0.001
	**Personal Characteristics**							
	Professional status	0.081	0.144	0.003				
	**Organizational Characteristics**							
	Type of organization	-0.166	-0.314	0.006				
Step 3					0.341	0.220	24.981	<0.001
	**Practice environment (PES-NWI Score)**	0.359	0.494	<0.001				

## Discussion

This study is one of the first that examined the job satisfaction of nurses who are caring for older adults in China, which enhances our understanding of nurses’ job satisfaction issues in elderly care institutions, and suggests that the nursing practice environment plays a critical role in explaining and predicting nurses’ job satisfaction in China.

The study found that the nurses who caring for older adults in Shanghai had moderate levels of job satisfaction. The findings are similar to the previous studies that focused on general nurses and correctional nurses; in China [34, 37] and the United States [38]. The satisfaction levels in this study are higher than the studies done in Ireland, where the study focus was on general nurses [39, 40].

Job satisfaction level in this study is slightly higher than the previous studies’ findings. Earlier studies show that nurses working in elderly care institutions had lower job satisfaction level than that of nurses in other types of working environments [41, 42]. Other than cultural and spatiotemporal differences, the differences between this study and the previous ones might also be explained by the distinct personnel structures and policy environment for nurses who caring for older adults in Shanghai.

According to the Chinese government’s “Eleventh Five-Year Plan” and the “Twelfth Five-Year Plan,” the Shanghai government had increased the number of nurses who are caring for older adults and improved their quality dramatically through greater emphasis on personal training. Firstly, nurses in elderly care institutions in Shanghai are young and well educated. As this study shows, more than a half of the nurses among elderly care institutions in Shanghai worked less than 9 years and most of them have a bachelor or higher degree, which is significantly higher than the China national average: 39.8% worked less than 9 years, and 8.8% had a bachelor or higher degree, according to the China health statistics yearbook 2011 [43]. Previous studies indicated that younger and unmarried nurses experienced less stress and had higher job satisfaction [38], and nurses having a higher education level may correspond with greater expertise and the desire to show self-awareness and self-worth on the job, which also attributed to higher job satisfaction [44]. Secondly, the Chinese government aims to improve healthcare equity and accessibility, so many high-quality medical institutions were set up in suburbs and high population growth areas in recent years [45]. In this context, more than a half of the nurses in this study were working in the institutions that are located in exurban and suburban areas, where the facilities are comparatively new with technologically advanced equipment, and have desirable nurse—patients’ ratios. All of the above mentioned reasons may explain the higher level of job satisfaction of nurses who caring for older adults.

This study explored the critical factors of nursing practice environment in explaining and predicting nurses’ job satisfaction in Shanghai, China.

Firstly, consistent with findings from other studies, this study also found that nurses who work in a favorable practice environment have higher level of job satisfaction compared with those work in an unfavorable or mixed environments [18, 46]. According to the PES-NWI, a favorable practice environment is characterized as more professional nurses who are able to interact with patients in a more authoritative, flexible and effective way. Favorable practice environments support nurses to mobilize resources quickly, cooperate with physicians effectively, and function at the highest level of clinical practice. Through these mechanisms, not surprisingly, favorable practice environments contribute to higher level of job satisfaction.

Secondly, although the positive relationship between nursing practice environment and RN job satisfaction has been well documented in hospital-based research [20, 47, 48], this study extended the line of inquiry into a transitional country’s elderly care institutions. In particular, as a critical factor, this study found that the practice environment characteristics had the strongest impact on nurses’ job satisfaction in elderly care institutions in Shanghai (Beta = 0.494).

As would be anticipated, this study showed that higher PES-NWI scores predict higher job satisfaction [49]. Although suggestions for improving work environments for nurses have been advanced by a number of nursing and regulatory organizations in other parts of the world [36], it is still rare in China. In addition, this study found that staffing and resource adequacy was the most unfavorable practice environment subscale, suggesting that future research is needed to assess factors related to the practice environment in China. New strategies are necessary for improving the nurses’ practice environment, including allocating enough staff, time, and resources for nurses who caring for older adults to provide quality care.

### Study limitations and recommendations for future research

The first limitation of the study is the cross-sectional design. Long-term follow-up studies are needed in order to explore the relationship between nurses’ job satisfaction and the practice environment in eldercare institutions. Second, this research is based on a questionnaire survey among institutional nurses who caring for older adults in Shanghai; although the study was conducted in a Chinese context, the findings presented may not be representative of China in general. There is a need to replicate such studies in other geographical areas in China. Third, when comparing the results of this study with others, available information on nurses’ job satisfaction among nurses in Chinese elderly care institutions is limited. Four, some studies have reported that depressive symptoms [50, 51] and burden of care [52, 53] could also play a role on job satisfaction. More severe depressive symptoms and higher burden of care may affect more on older nurses than the younger ones. More longitudinal, multi-regional, and multi-dimensional studies in the future can further verify the findings of this study.

## Conclusion

The job satisfaction model developed in this study suggests that the practice environment is a key predictor of nurses’ job satisfaction in China, which is similar to previous studies’ results. The new findings highlight the importance of the practice environment in eldercare institutions, since this has the strongest impact on nurses’ job satisfaction, outweighing any other variables and factors.

In order to improve nurses’ job satisfaction level and to make eldercare resources current and stable, a multi-dimensional strategy should be developed for addressing issues related to the practice environment in eldercare institutions and beyond.

## References

[1] Asadi A, Fadakar F, Khoshnodifar Z, Hashemi SM. Personal characteristics affecting agricultural extension workers' job satisfaction level. Journal of Social Sciences. 2008;4(4):246.

[2] Shields MA, Ward M. Improving nurse retention in the National Health Service in England: the impact of job satisfaction on intentions to quit. Journal of health economics. 2001;20(5):677–701. 11558644 10.1016/s0167-6296(01)00092-3

[3] Larrabee JH, Janney MA, Ostrow CL, Withrow ML, Hobbs GR Jr, Burant C. Predicting registered nurse job satisfaction and intent to leave. Journal of Nursing Administration. 2003;33(5):271 12792282 10.1097/00005110-200305000-00003

[4] Andrews DR, Dziegielewski SF. The nurse manager: job satisfaction, the nursing shortage and retention. Journal of Nursing Management. 2005;13(4):286–95. 15946168 10.1111/j.1365-2934.2005.00567.x

[5] China's Main Demographic Indicators from the 6th National Population Census [Internet]. 2011. Available from: http://www.stats.gov.cn/tjsj/pcsj/rkpc/6rp/indexch.htm.

[6] Microbell. The serious population aging in Chinese inland area microbell.com2012 [cited 2012 11–08]. Available from: http://www.microbell.com/ecodetail_714573.html.

[7] Xinhua. China's population aging highlight the shortage of professional nursing service xinhua network2012 [cited 2012 05–13]. Available from: http://news.xinhuanet.com/health/2012-05/13/c_123120045.htm.

[8] Kash BA, Naufal GS, Dagher RK, Johnson CE. Individual factors associated with intentions to leave among directors of nursing in nursing homes. Health care management review. 2010;35(3):246–55. 20551772 10.1097/HMR.0b013e3181dc826d

[9] Grant D. Physician financial incentives and cesarean delivery: new conclusions from the healthcare cost and utilization project. Journal of health economics. 2009;28(1):244–50. 10.1016/j.jhealeco.2008.09.005 19027184

[10] Manojlovich M. Linking the Practice Environment to Nurses' Job Satisfaction Through Nurse-Physician Communication. Journal of Nursing Scholarship. 2005;37(4):367–73. 16396411 10.1111/j.1547-5069.2005.00063.x

[11] Cimete G, Gencalp NS, Keskin G. Quality of life and job satisfaction of nurses. Journal of Nursing Care Quality. 2003;18(2):151 12680602 10.1097/00001786-200304000-00009

[12] Chan MF, Luk AL, Leong SM, Yeung SM, Van IK. Factors influencing Macao nurses’ intention to leave current employment. Journal of Clinical Nursing. 2009;18(6):893–901. 10.1111/j.1365-2702.2008.02463.x 19239668

[13] Kacel B, Miller M, Norris D. Measurement of nurse practitioner job satisfaction in a Midwestern state. Journal of the American Academy of Nurse Practitioners. 2005;17(1):27–32. 15679881 10.1111/j.1041-2972.2005.00007.x

[14] March JG, Simon HA. Organizations. 1958 NY: Wiley, New York. 1993.

[15] Ma CC, Samuels ME, Alexander JW. Factors that influence nurses' job satisfaction. Journal of Nursing Administration. 2003;33(5):293 12792284 10.1097/00005110-200305000-00005

[16] Upenieks VV. Assessing differences in job satisfaction of nurses in magnet and nonmagnet hospitals. Journal of Nursing Administration. 2002;32(11):564 12464774 10.1097/00005110-200211000-00004

[17] Burke RJ. Hospital restructuring, workload, and nursing staff satisfaction and work experiences. The Health Care Manager. 2003;22(2):99 12785546 10.1097/00126450-200304000-00003

[18] Zangaro GA, Soeken KL. A meta-analysis of studies of nurses' job satisfaction. Research in Nursing & Health. 2007;30(4):445–58. 17654483 10.1002/nur.20202

[19] Lake ET. Development of the practice environment scale of the nursing work index. Research in Nursing & Health. 2002;25(3):176–88. 12015780 10.1002/nur.10032

[20] Irvine DM, Evans MG. Job satisfaction and turnover among nurses: integrating research findings across studies. Nursing research. 1995. 7624236

[21] Caers R, Du Bois C, Jegers M, De Gieter S, De Cooman R, Pepermans R. Measuring community nurses' job satisfaction: literature review. J Adv Nurs. 2008;62(5):521–9. Epub 2008/03/22. 10.1111/j.1365-2648.2008.04620.x . 18355229

[22] Altier ME, Krsek CA. Effects of a 1-year residency program on job satisfaction and retention of new graduate nurses. Journal for Nurses in Staff Development. 2006;22(2):70 16603904 10.1097/00124645-200603000-00006

[23] Shields MA, Price SW. Racial harassment, job satisfaction and intentions to quit: evidence from the British nursing profession. Economica. 2002;69(274):295–326.

[24] Happell B, Martin T, Pinikahana J. Burnout and job satisfaction: A comparative study of psychiatric nurses from forensic and a mainstream mental health service. International journal of mental health nursing. 2003;12(1):39–47. 14685958 10.1046/j.1440-0979.2003.00267.x

[25] Iskera-golec I, Folkard S, Marek T, Noworol C. Health, well-being and burnout of ICU nurses on 12-and 8-h shifts. Work & Stress. 1996;10(3):251–6.

[26] Wu B, Mao Z, Xu Q. Institutional care for elders in rural China. Journal of aging & social policy. 2008;20(2):218–39. 18788366 10.1080/08959420801977632

[27] Feng Z, Liu C, Guan X, Mor V. China’s rapidly aging population creates policy challenges in shaping a viable long-term care system. Health Affairs. 2012;31(12):2764–73. 10.1377/hlthaff.2012.0535 23213161 PMC3711106

[28] Hong T, Zhonghua Y, Sheng Y, Yi G, Fangjun W, Lihua Z, et al Study on Hospitalization Services Utilizaztion and Its Influencing Factors in Elderly in Nanhui District of Shanghai. Chinese Primary Health Care. 2010;24(10):17–9.

[29] Yang Y. Research on Status and Policies of Nursing Care for the Aged in Shanghai: Fudan University; 2011.

[30] Ministry of Health of the People's Republic of China. Issued a circular on "China's guidelines to community healthcare service centers setiing " notice The web of the Central People's Government of the People's Republic of China: inistry of Health of the People's Republic of China; 2006 [cited 2006 2006-08-18]. Available from: http://www.gov.cn/zwgk/2006-09/04/content_377067.htm.

[31] Zangaro GA, Soeken KL. Meta-analysis of the reliability and validity of Part B of the Index of Work Satisfaction across studies. Journal of nursing measurement. 2005;13(1):7–22. 16315567 10.1891/jnum.2005.13.1.7

[32] Stamps PL. Nurses and work satisfaction: an index for measurement: Health Administration Press; 1997.

[33] Gardner JK, Thomas-Hawkins C, Fogg L, Latham CE. The relationship between nurses' perceptions of the hemodialysis unit work environment and nurse turnover, patient satisfaction, and hospitalizations. Nephrology Nursing Journal. 2007;34(3):271 17644871

[34] Wu L. Study of Work Satisfaction and Turn over Intention among Chinese nurse in Changsha. Changsha: Central South University; 2007.

[35] Zheng X. Nurse Job Satisfaction and its Influencing Factors in 3-A-grade General Hospitals in Beijing: Chinese Peking Union Medical College; 2009.

[36] Patrician PA, Shang J, Lake ET. Organizational determinants of work outcomes and quality care ratings among Army Medical Department registered nurses. Research in nursing & health. 2010;33(2):99–110. 20151409 10.1002/nur.20370 PMC2969846

[37] Zeng Q. Study of work satisfaction and turnover intention among headnurses in changsha city Central South University; 2008.

[38] Flanagan NA, Flanagan TJ. An analysis of the relationship between job satisfaction and job stress in correctional nurses. Research in nursing & health. 2002;25(4):282–94. 12124722 10.1002/nur.10042

[39] Curtis E. Job satisfaction: a survey of nurses in the Republic of Ireland. International nursing review. 2007;54(1):92–9. 17305963 10.1111/j.1466-7657.2007.00507.x

[40] Karanikola MN, Papathanassoglou EDE, Giannakopoulou M, Koutroubas A. Pilot exploration of the association between self-esteem and professional satisfaction in Hellenic Hospital nurses. Journal of Nursing Management. 2007;15(1):78–90. 17207011 10.1111/j.1365-2934.2006.00673.x

[41] Carr KK, Kazanowski MK. Factors affecting job satisfaction of nurses who work in Long-term care. Journal of Advanced Nursing. 1994;19(5):878–83. 8056916 10.1111/j.1365-2648.1994.tb01164.x

[42] Yu H, Sheng z. Investigation on the training status of older adults' nursing staff in a local city. Chinese Journal of Nursing Education. 2012;9(7):319–22.

[43] China MoHo. China health statistics yearbook 2011 Web of Ministry of Health of China: Ministry of Health of China; 2013 [cited 2013 01–16]. Available from: http://61.49.18.65/htmlfiles/zwgkzt/ptjnj/year2011/index2011.html.

[44] Afroz N, Mittra R. Higher education and professionalism: through the lens of self-actualizing. Dimensions of Education. 2010:137–9.

[45] Government SMPs. A five-year plan(from 2011 to 2015) for Shanghai health reform and development Shanghai.gov2012 [cited 2012 02–20]. Available from: http://www.shanghai.gov.cn/shanghai/node2314/node2319/node2404/n29419/n29420/u26ai31145.html.

[46] Hall DS. The relationship between supervisor support and registered nurse outcomes in nursing care units. Nursing Administration Quarterly. 2007;31(1):68–80. 17198122 10.1097/00006216-200701000-00015

[47] Choi Sandy Pin-Pin Bsn RN, Cheung Kin PhD RN, Pang Samantha Mei-Che PhD RN. Attributes of nursing work environment as predictors of registered nurses’ job satisfaction and intention to leave. Journal of Nursing Management. 2013;21(3):429–39. 10.1111/j.1365-2834.2012.01415.x 23409781

[48] Manojlovich M, Laschinger HKS. The relationship of empowerment and selected personality characteristics to nursing job satisfaction. Journal of Nursing Administration. 2002;32(11):586–95. 12464776 10.1097/00005110-200211000-00006

[49] Friese CR, editor Nurse practice environments and outcomes: implications for oncology nursing Oncology nursing forum; 2005: Onc Nurs Society. 10.1188/05.ONF.765-772 15990906

[50] Ruggiero JS. Health, work variables, and job satisfaction among nurses. Journal of Nursing Administration. 2005;35(5):254–63. 15891489 10.1097/00005110-200505000-00009

[51] Petterson I-L, Arnetz B, Arnetz J. Predictors of job satisfaction and job influence–results from a national sample of Swedish nurses. Psychotherapy and Psychosomatics. 1995;64(1):9–19. 7480584 10.1159/000288986

[52] Cohen JD. The aging nursing workforce: how to retain experienced nurses. Journal of healthcare management. 2006;51(4):233 16916117

[53] FILLION L, DUPUIS R, TREMBLAY I, DE GRÂCE G-R, BREITBART W. Enhancing meaning in palliative care practice: a meaning-centered intervention to promote job satisfaction. Palliative & supportive care. 2006;4(04):333–44. 17133893 10.1017/s1478951506060445

